# Transgenic rat with ubiquitous expression of angiotensin-(1-7)-producing fusion protein: a new tool to study the role of protective arm of the renin-angiotensin system in the pathophysiology of cardio-renal diseases

**DOI:** 10.1038/s41440-024-01995-y

**Published:** 2024-11-13

**Authors:** Luděk Červenka, Zuzana Husková, Soňa Kikerlová, Olga Gawrys, Šárka Vacková, Petra Škaroupková, Janusz Sadowski, Matúš Miklovič, Matej Molnár, Miloš Táborský, Vojtěch Melenovský, Michael Bader

**Affiliations:** 1 Center for Experimental Medicine, Institute for Clinical and Experimental Medicine, Prague, Czech Republic; 2 Department of Internal Medicine I, Cardiology, University Hospital Olomouc and Palacký University, Olomouc, Czech Republic; 3 Department of Physiology, Faculty of Science, Charles University, Prague, Czech Republic; 4 Department of Pathophysiology, Second Faculty of Medicine, Charles University, Prague, Czech Republic; 5 Department of Cardiology, Institute for Clinical and Experimental Medicine, Prague, Czech Republic; 6 Max-Delbrück Center for Molecular Medicine in the Helmholtz Association (MDC), Berlin, Germany; 7 DZHK (German Center for Cardiovascular Research), Partner Site Berlin, Berlin, Germany; 8 Institute for Biology, University of Lübeck, Lübeck, Germany; 9 Charité University Medicine Berlin, Berlin, Germany

**Keywords:** Renin-angiotensin system, Angiotensin II, Angiotensin-(1-7), TG7371 transgenic rat

## Abstract

The aim of the present study was to assess systemic circulatory and tissue activities of both the classical arm and of the alternative arm of the renin-angiotensin system (RAS) in a new transgenic rat line (TG7371) that expresses angiotensin-(1-7) (ANG 1-7)-producing fusion protein; the results were compared with the activities measured in control transgene-negative Hannover Sprague-Dawley (HanSD) rats. Plasma and tissue concentrations of angiotensin II (ANG II) and ANG 1-7, and kidney mRNA expressions of receptors responsible for biological actions of ANG II and ANG 1-7 [i.e. ANG II type 1 and type 2 (AT_1_ and AT_2_) and Mas receptors] were assessed in TG7371 transgene-positive and in HanSD rats. We found that male TG7371 transgene-positive rats exhibited significantly elevated plasma, kidney, heart and lung ANG 1-7 concentrations as compared with control male HanSD rats; by contrast, there was no significant difference in ANG II concentrations and no significant differences in mRNA expression of AT_1_, AT_2_ and Mas receptors. In addition, we found that in male TG7371 transgene-positive rats blood pressure was lower than in male HanSD rats. These data indicate that the balance between the classical arm and the alternative arm of the RAS was in male TGR7371 transgene-positive rats markedly shifted in favor of the latter. In conclusion, TG7371 transgene-positive rats represent a new powerful tool to study the long-term role of the alternative arm of the RAS in the pathophysiology and potentially in the treatment of cardio-renal diseases.

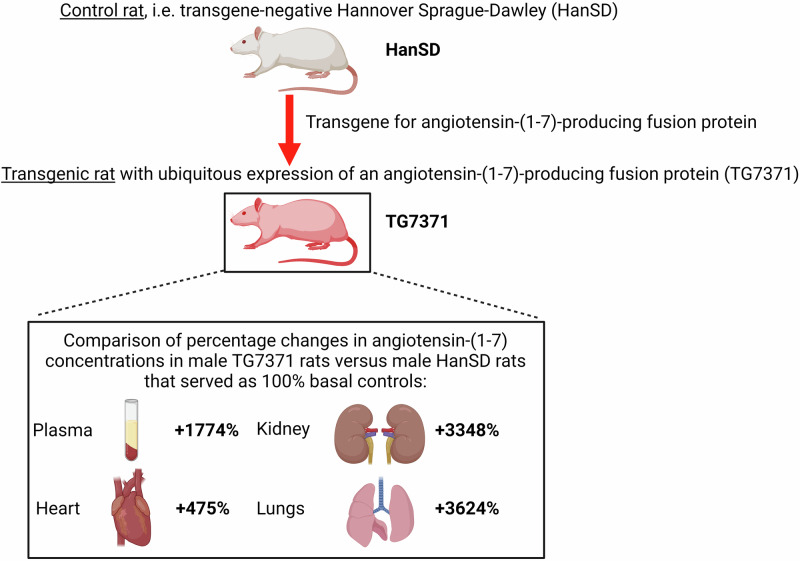

## Introduction

The critical role of the renin-angiotensin system (RAS) in the regulation of sodium balance, extracellular fluid volume, renal hemodynamics and tubular function and renal and systemic vascular resistance has been recognized for decades [1–3]. Inappropriate activation of this powerful system and especially its long-term stimulation makes RAS the major factor contributing to the development of hypertension, progression of heart failure (HF) and chronic kidney disease (CKD) [1–8]. RAS activation may at first help compensate some pathophysiological abnormalities and is initially beneficial but in the long run, it is clearly deleterious. The classic arm of the RAS consists of the enzymes renin and angiotensin-converting enzyme (ACE) which ultimately generate angiotensin II (ANG II) [in two steps, first renin forms angiotensin I (ANG I) from angiotensinogen and then ANG I is converted by ACE to ANG II]. Activation by ANG II of type 1 receptors (AT_1_) is considered to be responsible for all physiological as well as pathophysiological actions of the RAS [1, 4, 5, 8]. Therefore, in current guideline-directed medical therapy related to the RAS, pharmacological blockade of this axis either by ACE inhibitors (ACEi) or AT_1_ receptor antagonists is the golden standard for the treatment of hypertension, HF and CKD [9–11].

However, 36 years ago it was discovered that in the brainstem homogenates of dogs ANG II was converted along an ACE-independent pathway to a heptapeptide angiotensin-(1-7) (ANG 1-7) [12]. This gave rise to a hypothesis on the biological function of this peptide in the brain [13]. Further research by many groups established the existence of the second, so called alternative, axis of the RAS, which is based on ACE type 2 (ACE2). This axis is responsible for ANG 1-7 formation that activates G-protein-coupled receptor Mas; it is thought that this alternative axis counteracts the classical arm of the RAS [14–16].

The classical arm, with ANG II as the main biologically active peptide, mediates vasoconstrictor, sodium retaining, inflammatory, proliferative and profibrotic actions; when inappropriately activated this axis is responsible for the pathophysiological actions of the RAS in cardio-renal diseases [17, 18]. The second (alternative) RAS axis, with ANG 1-7 as the main biologically active peptide, induces vasodilatation, natriuresis, and exerts anti-inflammatory, anti-hypertrophic and anti-fibrotic actions. Its activation could serve as endogenous physiological tool to antagonize deleterious actions of inappropriately increased ANG II concentration [4, 15, 16].

It is postulated that activation of ACE2/ANG 1-7/Mas arm of the RAS, and strictly augmentation of ANG 1-7 concentrations, could be a novel therapeutic tool for cardio-renal diseases, particularly hypertension, HF and CKD [14–16]. To be effective, long-term augmentation of ANG 1-7 levels should be achieved. Since ANG 1-7 is known to function primarily at the tissue level rather than as a circulating hormone, a long-term increase in organs would be required [15, 16]. This was attempted with chronic infusion of ANG 1-7 using osmotic minipumps. To evaluate long term ANG 1-7 effects on animals’ morbidity and mortality in HF and CKD, repeated implantation of minipumps is required and this approach still does not consistently increase tissue ANG 1-7 [15, 16]. Therefore, to overcome these limitations a transgenic rat strain was generated that expresses an ANG 1-7-producing fusion protein in the testis [19]. The testis operates here as an endogenous pump for ANG 1-7 infusion. Indeed, plasma ANG 1-7 was reported to be 4.5-fold higher than in transgene-negative Hannover Sprague-Dawley (HanSD) rats, however, in the kidney, heart, lung and brain the levels were not elevated [19]. Moreover, in a later study using this transgenic rat model we did not find increased organ ANG 1-7 concentrations, nor was there any elevation of circulating ANG 1-7 levels [20]. Evidently, this model proved, again, unsuitable for investigation of long-term effects of increased tissue ANG 1-7 concentrations. Therefore, a new transgenic rat strain with expression of an ANG 1-7-producing fusion protein in all tissues was recently developed [strain name: (TG7371)]. Since it was demonstrated that the transgene-specific mRNA and the corresponding fusion protein were present in all evaluated tissues [21], it was concluded that this is, indeed, a model with ubiquitous expression of ANG 1-7-producing fusion protein [21]. It was shown that plasma ANG 1-7 levels in TG7371 were not significantly different from the values observed in HanSD rats but were about 50% higher in the hypothalamus; there were no significant alterations in plasma and hypothalamic ANG II concentrations [21]. These findings suggested that TG7371 is a model with increased tissue production of ANG 1-7 with little or no spillover to the circulation and with unaltered ANG II concentrations. Thus, it is proposed that TG7371 is a good animal model to further explore the long-term impact of increased tissue ANG 1-7 levels on the pathophysiology of cardio-renal diseases [21]. However, before making an ultimate conclusion that TG7371 is an optimal model to study the role ACE2/ANG 1-7/MAS arm in the pathophysiology of cardio-renal diseases, and before exploration of the possible benefit of activation of this axis in hypertension, HF and CKD, some limitations in the characterization of the model should be indicated.

First, the characterization study was based on the assessment of mRNA expression coding for the ANG 1-7-producing fusion protein in multiple tissues, whereas the actual ANG 1-7 levels were determined in the hypothalamus only [21].

The second limitation is that phenotyping did not include female TG7371 rats. Given the known sex-linked differences in the pathophysiology of cardiovascular diseases [22–25], “sex” should no longer be an ignored experimental variable, being an unavoidable parameter in preclinical research and the prerequisite for successful translation of results into clinical practice [26]. Importantly, there are important sex-linked differences between the classical arm and alternative arm of the RAS [27].

Therefore, the first aim of the present study was to determine circulating and tissue concentrations of ANG 1-7, the marker of activity of the alternative arm of the RAS, and ANG II concentrations, the marker of the classical arm of the RAS, in male and female TG7371 and HanSD rats.

The second aim of the present study was to obtain a more detailed insight into the effects of the possibly long-term increased ANG 1-7 levels on the alternative and classical arms of the RAS. Kidney mRNA expressions of ACE2 and receptors responsible for biological actions of ANG II and ANG 1-7 [i.e. AT_1_, ANG II type 2 (AT_2_) and Mas receptors] were assessed in male and female TG7371 and HanSD rats.

Finally, the third aim of the present study was to examine if relatively long increases in ANG 1-7 levels would significantly alter cardiovascular phenotype, i.e. decrease blood pressure (BP) in male and female TG7371 rats as compared with age-matched HanSD rats.

## Methods

### Ethical approval and animals

The study was performed in accordance with the guidelines and practices established by the Animal Care and Use Committee of the Institute for Clinical and Experimental Medicine (IKEM), Prague, which accord with the European Convention on Animal Protection and Guidelines on Research Animal Use, and were approved by this committee and subsequently by the Ministry of Health of the Czech Republic (the decision number for this project is 17078/2022-5/OVZ). All animals used in the study were bred in IKEM, which is accredited by the Czech Association for Accreditation of Laboratory Animals. Heterozygous breeder pairs of TG7371 were obtained from Max Delbrück Center for Molecular Medicine, Berlin, Germany. HanSD rats were also originally obtained from Max Delbrück Center.

## Detailed experimental design

### General animal specification

Three different groups of male and female animals were examined. The first group consisted of HanSD rats and served as overall control animals. The second group consisted of TG7371 transgene-negative rats and served also as a control group; the genotype was confirmed by quantitative real-time reverse polymerase chain reaction (PCR) as described below. The reason for choosing TG7371 transgene-negative rats as the second control group was to examine if the long-term breeding of heterozygous TG7371 (with potential variability of transgene expression among TG7371 transgene-positive rats in the long-term perspective) does not alter the phenotype, particularly activity of the RAS, in transgene-negative rats. The third group consisted of heterozygous TG7371 transgene-positive rats and served as the strain in which overactivation of the alternative arm of the RAS should be present.

At the appropriate time point (i.e. at the age of 14 weeks), the samples for measurement of plasma and organ ANG II and ANG 1-7 concentrations were obtained from conscious decapitated rats (NB.: it was established that anesthesia alters concentration of these peptides [28–32]).

### Measurement of plasma and tissue ANG II and ANG 1-7 concentrations

Preparation of blood samples: immediately after decapitation, whole blood was collected into cold tubes containing 250 μl of inhibitor cocktail (5 mM EDTA, 1.25 mM 1,10-phenanthroline, 20 μM enalapril maleate, 10 μM pepstatin A), kept on ice (rocked) and then centrifuged at 4 °C and 3000 g for 10 min to separate the plasma. After centrifugation, 1 ml (for ANG II) and 2 ml (for ANG 1-7) of plasma was precipitated with 4 ml of ethanol and again centrifuged at 4 °C and 3000 g for 10 min. The supernatant was evaporated using a vacuum centrifuge (Savant SpeedVac). Dried samples were stored at −20 °C or below until assayed.

Preparation of tissue samples: immediately after decapitation and blood collection, the kidneys (and other organs) were removed, dried, weighed and 0.5 g of the tissue was homogenized in 3 ml pre-cooled methanol. In the case of the heart, after removal it was dried, weighed, divided into atria, left ventricle + septum and right ventricle, and 0.5 g of the left ventricle tissue was homogenized in 3 ml pre-cooled methanol. Analogous procedures were used for lung tissue. The tube with homogenate was kept on ice and then centrifuged at 4 °C and 3000 g for 10 min. The supernatant was evaporated using Savant SpeedVac vacuum centrifuge. Dried samples were stored at −20 °C or below until solid-phase extraction (for kidney samples) and the assay. For the determination of ANG II, kidney homogenates were purified by solid-phase extraction. Dried kidney samples were reconstituted with 4 ml of 50 mM sodium phosphate buffer (pH 7.4) containing 267 mg bovine serum albumin/l and kept on ice. Phenyl-bonded solid phase extraction columns (SPE) (Bond-Elut®PH, Agilent) were preconditioned with methanol (3 ml), followed by distilled-water (2 × 3 ml). Thereafter, reconstituted kidney samples were applied to pre-washed columns. The columns were sequentially washed with distilled-water (3 ml), hexane (3 ml) and chloroform (3 ml); water removes salts and other polar substances from the columns. Hexane and chloroform elute contaminating lipids and hydrophobic material from the columns but do not affect angiotensin peptides recovery. At the end, angiotensin peptides were eluted from SPE columns using 2 × 1 ml flush of methanol. Similarly, for the determination of ANG 1-7, dried plasma and kidney samples were reconstituted with 4 ml of 50 mM sodium phosphate buffer (pH 7.4) containing 267 mg BSA/l and kept on ice. C18-bonded SPE columns (Bond-Elut®C18, Agilent) were preconditioned with mixture of ethanol + distilled-water + 4% acetic acid (83:13:4 by volume; 5 ml), methanol (5 ml), distilled-water (5 ml) and with 4% acetic acid (5 ml). Thereafter, reconstituted samples were applied to pre-washed columns. The columns were sequentially washed with distilled-water (5 ml) and acetone (5 ml). At the end, ANG peptides were eluted from SPE columns with 2 × 1 ml + 1 × 1.5 ml of mixture of ethanol + distilled-water + 4% acetic acid (83:13:4 by volume). The eluates were evaporated to dryness using a vacuum centrifuge. Dried samples were stored at −20 °C or lower until assayed. ANG II levels were measured by a competitive radioimmunoassay, using the commercially available RIA kit (Catalog number ED29051, IBL International, Hamburg, Germany). ANG 1-7 levels were measured also by competitive RIA using the custom-made RIA kit (Immunotech s.r.o., Prague, Czech Republic). All these methods are routinely employed in our laboratory [28–32].

### RNA isolation and quantitative real-time PCR (qRT-PCR) for mRNA of ANG 1-7-producing fusion protein

As mentioned above, after decapitation the kidney samples were snap-frozen in liquid nitrogen and stored at −80 °C for further analysis. Approximately 100 mg of kidney cortex was used for analysis. Total RNA was isolated with RNAzol® RT (Sigma-Aldrich®) according to the manufacturer’s instructions. The amount of isolated total RNA in each sample was measured by DeNovix Spectrophotometer (DS-11 Series, DeNovix Inc.) and subsequently diluted to yield 100 ng in each sample. qRT-PCR analysis was performed with One Step TB Green® PrimeScript^TM^ RT-PCR Kit II (Catalog number RR08A, Takara Bio Inc.) and analyzed with ViiA™ 7 Real-time PCR system (Applied Biosystems) according to manufacturer’s instructions. In all experiments, relative gene expression was calculated by the 2 − ∆Ct method, which is most frequently used for such experiments and is also employed in our laboratory [33–35]. This method directly uses the Ct (threshold cycle) information generated from a qPCR system. Transgene-specific primers were as follows: *forward primer* IG5 (5′-TTCCTTCTCATGCAACGTGA-3′) located in IgG fragment and the *reverse primer* hRENEX (5′-CTTCAGGCTTTCTCGGATTG-3′) located in the human prorenin fragment of the transgene. To calculate relative gene expression in target and reference samples, we employed the GAPDH gene as a housekeeping gene and used it as the normalizer because its expression level remains relatively stable. First, we calculated ΔCt of each sample following the formula:

$$\Delta {{{\rm{Ct}}}}={{{\rm{Ct}}}}({{{\rm{gene}}}}\; {{{\rm{of}}}}\; {{{\rm{interest}}}})-{{{\rm{Ct}}}}({{{\rm{housekeeping}}}}\; {{{\rm{gene}}}})$$

The expression of mRNA of selected genes was related to that in the control group i.e. HanSD rats. The final results were expressed as the n-fold difference in gene expression of mRNA of target genes between the appropriate experimental group and control group calculated as follows:

$${{{\rm{n}}}}-{{{\rm{fold}}}}\; {{{\rm{expression}}}}=	 2-(\Delta {{{\rm{Ct}}}}\; {{{\rm{of}}}}\; {{{\rm{the}}}}\; {{{\rm{experimental}}}}\; {{{\rm{group}}}}\\ 	-\Delta {{{\rm{Ct}}}}\; {{{\rm{of}}}}\; {{{\rm{the}}}}\; {{{\rm{control}}}}\; {{{\rm{group}}}})$$

Subsequently, the log transformation of the data was performed to make it more symmetrical, as recommended and generally accepted for evaluation of relative gene expression results. Thus, the values in the graphs represent log2 n-fold gene expression.

After confirming the transgene-specific mRNA in the kidney tissue, animals were divided into the following groups (the analyses of organ weights, and ANG II and ANG 1-7 concentrations were done using the blind procedure):

1. Male HanSD rats (*n* = 14 in plasma and all organs)
2. Male TG7371 transgene-negative rats (*n* = 10–12 depending on the organ)
3. Male TG7371 transgene-positive rats (*n* = 9–13 depending on the organ)
4. Female HanSD rats (*n* = 16 in plasma and all organs)
5. Female TG7371 transgene-negative rats (*n* = 8–9 depending on the organ)
6. Female TG7371 transgene-positive rats (*n* = 6–7 depending on the organ)

The specific n for each experimental group of male and female TG7371 transgene-negative and transgene-positive rats and specific organ are always given at the appropriate results section.

### Assessment of kidney mRNA expression for ACE2, AT1, AT2 and Mas receptors

Measurement of multiple mRNA was performed by the standard technique described above. The measurement of multiple mRNA expression was performed in accordance with the manufacturer’s instructions (384‐well microfluidics TaqMan array cards; custom setting of selected genes; Applied Biosystems, Foster City, CA, USA). The genes, which were investigated are listed below, including appropriate ID assay identification number, and were evaluated in our recent studies [34, 35]: ACE2 – ID Assay Number: Rn01416293_m1, AT_1A_ receptor - ID Assay Number: Rn02758772_s1, AT_2_ receptor - ID Assay Number: Rn00560677_s1, Mas receptor - ID Assay Number: Rn00562673_s1.

### Assessment of cardiovascular phenotype, i.e. systolic BP (SBP) development

SBP was measured with automated tail-cuff system (Hatteras Instruments, Cary, N.C., USA). Currently, two approaches for BP measurement are used, either invasive radiotelemetry or the noninvasive tail-cuff technique. Both methods have advantages and disadvantages as emphasized in recommendation for BP measurements in experimental animals that was published 20 years ago in the American Heart Association statement [36]. More recently there has been increasing perception that the tail-cuff BP measurements is not an acceptable approach for BP in experimental animals. On the other hand, the editors of *Hypertension* stated recently their assurance that noninvasive tail-cuff measurements of BP is still fully acceptable in many instances and appropriate recommendations were formulated [37]. Indeed, our earlier studies have shown a close correlation between measurements by tail-plethysmography and direct BP measurements using either radiotelemetry or indwelling catheter [38–41], and similar rigorous correlation was also found by other investigators [42, 43]. Admittedly, the tail-cuff method does not allow accurate measurements of diastolic BP and mean arterial pressure, but the method still provides valuable information regarding BP differences between groups over long time.

At the age of 8 weeks accustom training for indirect tail-cuff SBP procedure was started as we described previously [38–40]: the animals were trained 3 days per week over a period of two weeks. The actual SBP measurement was started at the age of 10 weeks and was also performed on the 12th and 14th week of age. In the meantime, i.e. on weeks 11th and 13th of age the animals were again exposed to the accustomed procedure. The presented value of SBP is an average from 4 measurements per one session and the person (P.Š.) who did the actual SBP measurement was unaware of the identity of the group in the study (groups were labeled A, B and C). On the day of SBP measurements, the body weight of animals was also assessed. The following groups were examined:

1. Male HanSD rats (*n* = 11)
2. Male TG7371 transgene-negative rats (*n* = 11)
3. Male TG7371 transgene-positive rats (*n* = 11)
4. Female HanSD rats (*n* = 12)
5. Female TG7371 transgene-negative rats (*n* = 9)
6. Female TG7371 transgene-positive rats (*n* = 9)

### Statistical analysis

All values are expressed as means ± SEM. Using the Graph-Pad Prism software (Graph Pad Software, San Diego, CA, USA), statistical analysis was done by one-way analysis of variance (ANOVA) followed by Tukey-Kramer test (multiple comparisons test was used to compare values between individual experimental groups). The values exceeding 95% probability limits (*p* < 0.05, two-sided) were considered statistically significant.

## Results

### Confirmation of kidney expression of mRNA for the ANG 1-7-producing fusion protein by qRT-PCR and effects on body and organ weights

**Fig. 1:**
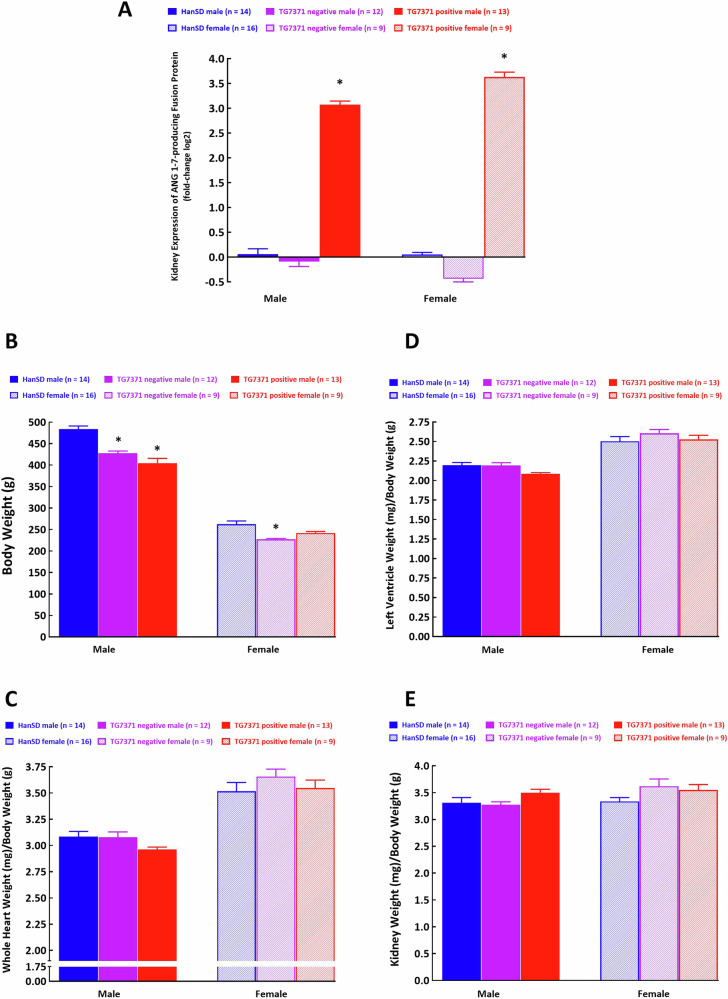
**A** Kidney mRNA expression of angiotensin-(1-7) (ANG 1-7)-producing fusion protein, **B** body weight, **C** whole heart weight, **D** left ventricle weight and **E** whole kidney weight normalized to body weight in control Hannover Sprague-Dawley (HanSD) rats (shown in blue), in transgene-negative rats for ANG 1-7-producing fusion protein (TG7371 negative) (violet) and in transgene-positive rats for ANG 1-7-producing fusion protein (TG7371 positive) (in red). Data for males are shown as intense-colored bars and for females as light-colored bars. ^*^P < 0.05 compared with HanSD rats, always in the same sex
- *Body weight and organ weights*
  Body weight in male TG7371 transgene-negative rats as well as in TG7371 transgene-positive rats was significantly lower than in control HanSD rats (Fig. 1B). In the case of female rats, only TG7371 transgene-negative animals showed body weight significantly lower than observed in HanSD controls; also in female transgene-positive TG7371 the body weight tended to be lower albeit without statistically significant difference (Fig. 1B). There were no significant differences in whole heart weight, left ventricle weight and kidney weight when normalized to body weight in male TG7371 transgene-positive, male TG7371 transgene-negative and in control male HanSD rats, and the same was true for female animals (Fig. 1C–E).

- *Kidney expression of ANG 1-7-producing fusion protein*
  Kidney mRNA expression for the ANG 1-7-producing fusion protein in male as well as female TG7371 transgene-positive rats was very high. Male as well as female HanSD rats and TG7371 transgene-negative rats showed no mRNA expression for ANG 1-7-producing fusion protein in kidney tissue, as expected (Fig. 1A). Based on these data, all the other parameters measured, such as body and organ weights and ANG 1-7 and ANG II concentrations, were allocated to appropriate groups.

### Effects of expression of an ANG 1-7-producing fusion protein on plasma and tissue ANG 1-7 and ANG II concentrations

**Fig. 2:**
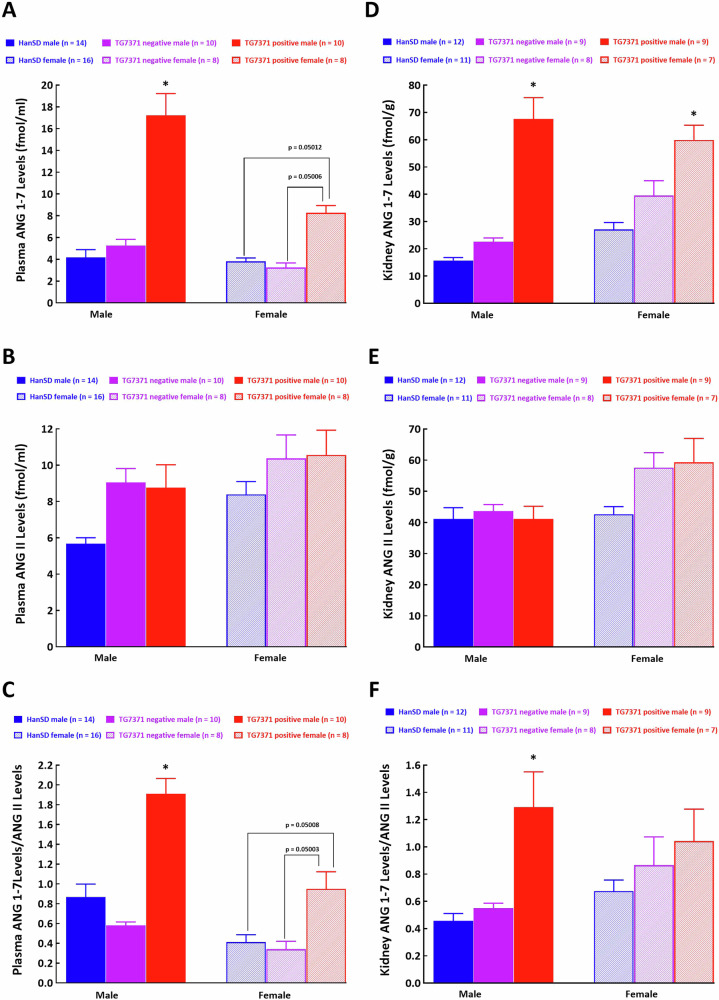
**A** Plasma angiotensin-(1-7) (ANG 1-7) levels, **B** plasma angiotensin II (ANG II) levels, **C** plasma ANG 1-7 to ANG II ratio, **D** kidney ANG 1-7 concentrations, **E** kidney ANG II concentrations and **F** kidney ANG 1-7 to ANG II ratio in control Hannover Sprague-Dawley (HanSD) rats (shown in blue), in transgene-negative rats for ANG 1-7-producing fusion protein (TG7371 negative) (violet) and in transgene-positive rats for ANG 1-7-producing fusion protein (TG7371 positive) (red). Data for males are shown as intense-colored bars and for females as light-colored bars. ^*^P < 0.05 compared with HanSD rats, always in the same sex
- *Kidney ANG 1-7 and ANG II concentrations*
  Kidney ANG 1-7 concentrations in male as well as in female TG7371 transgene-positive rats were significantly higher than in male and female HanSD controls (Fig. 2D), again, in the absence of differences in kidney ANG II levels (Fig. 2E). However, in male TG7371 transgene-positive rats the ANG 1-7/ANG II ratio increased about 3-fold when compared with male HanSD controls, but it was not increased in female TG7371 transgene-positive rats compared with female HanSD controls (Fig. 2F).
- *Lung ANG 1-7 and ANG II concentrations*
  Lung ANG 1-7 concentrations in male as well as in female TG7371 transgene-positive rats were about 40-fold and 30-fold higher than, respectively, in male and female HanSD controls (Fig. 3A). Again, there were no differences in ANG II concentrations (Fig. 3B), which resulted in about 20-fold and 15-fold increase, respectively, in ANG 1-7/ANG II ratio, both in in male and female TG7371 transgene-positive rats when compared with their HanSD counterparts (Fig. 3C).

- *Plasma ANG 1-7 and ANG II levels*
  Plasma ANG 1-7 levels in male TG7371 transgene-positive rats were about 4-fold higher than in male control HanSD rats (Fig. 2A), in the absence of differences in plasma ANG II levels (Fig. 2B). This resulted in a marked increase in the ratio of ANG 1-7 to ANG II in plasma, indicating domination of the alternative axis of the RAS (Fig. 2C). This ratio has been validated as a reliable marker for evaluation of the activity of ACE2/ANG 1-7 axis [30] and it is employed by our laboratory when the activity of both axes of the RAS under various pathophysiological situation is assessed [20, 29–31, 41, 44]. Plasma ANG 1-7 and ANG II levels in female TG7371 transgene-positive rats were not significantly different from the values observed in female HanSD controls (Fig. 2A, B). Even though there was a tendency for higher plasma values of ANG 1-7 in female TG7371 transgene-positive rats as compared with female HanSD rats and female TG7371 transgene-negative rats (*p* = 0.05012 as compared with female HanSD rats and *p* = 0.05006 as compared with female TG7371 transgene-negative rats, respectively), evidently the difference did not reach statistical significance level (Fig. 2A). The tendency for higher ANG 1-7/ANG II ratio in female TG7371 transgene-positive rats as compared with female HanSD rats and female TG7371 transgene-negative rats was even more obvious but, again, the difference did not reach statistical significance level (*p* = 0.05008 as compared with female HanSD rats and *p* = 0.05003 as compared with female TG7371 transgene-negative rats, respectively), (Fig. 2C).

**Fig. 3:**
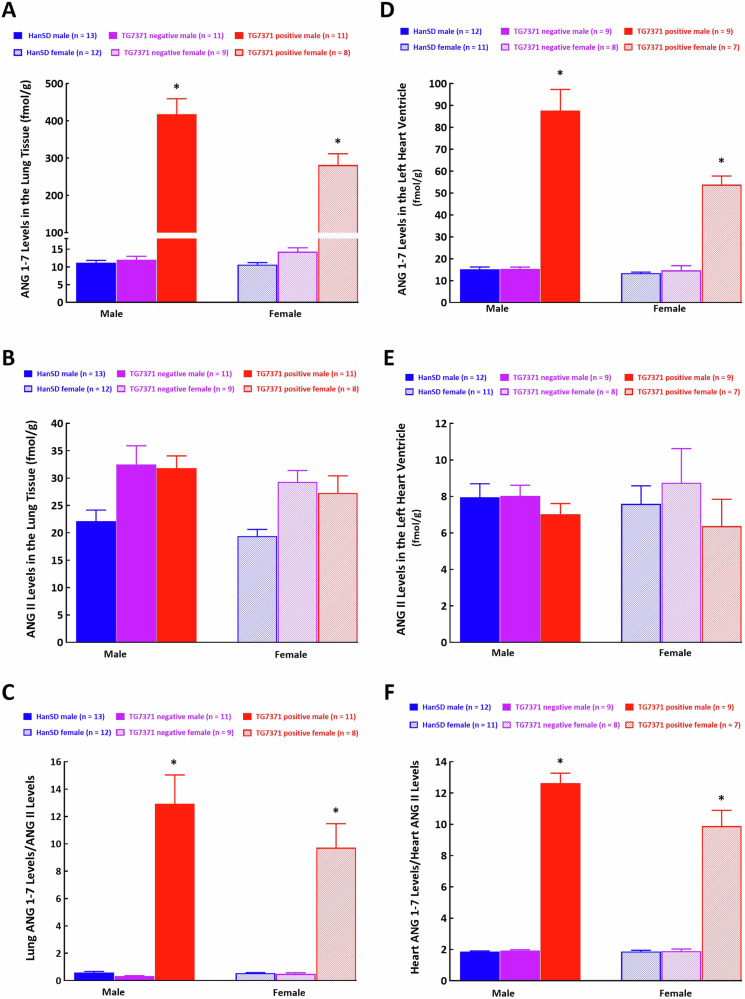
**A** Angiotensin-(1-7) (ANG 1-7) concentrations in lung tissue, **B** angiotensin II (ANG II) concentrations in lung tissue, **C** lung ANG 1-7 to ANG II ratio, **D** ANG 1-7 concentrations in the left heart ventricle, **E** ANG II concentrations in the heart left ventricle and **F** heart left ventricle ANG 1-7 to ANG II ratio in control Hannover Sprague-Dawley (HanSD) rats (shown in blue), in transgene-negative rats for ANG 1-7-producing fusion protein (TG7371 negative) (violet) and in transgene-positive rats for ANG 1-7-producing fusion protein (TG7371 positive) (red). Data for males are shown as intense-colored bars and for females as light-colored bars. ^*^P < 0.05 compared with HanSD rats, always in the same sex
- *Left ventricle (LV) ANG 1-7 and ANG II concentrations*
  LV heart ANG 1-7 concentrations in male and female TG7371 transgene-positive rats were about 5-fold and 3-fold higher than in male and female HanSD controls, respectively (Fig. 3D). Again, there were no differences in ANG II concentrations (Fig. 3E), which resulted in about 6-fold and 4-fold increase in ANG 1-7/ANG II ratio, respectively, both in in male and female TG7371 transgene-positive rats when compared with their HanSD counterparts (Fig. 3F).
  Collectively, male as well as female TG7371 transgene-negative rats did not show any significant differences in plasma and organ ANG 1-7 and ANG II concentrations as compared with male and female HanSD controls (Figs. 2 and 3).
- *Overview of percentage changes in ANG 1-7 concentrations in male and female TG7371 transgene-positive rats versus HanSD rats*

**Fig. 4:**
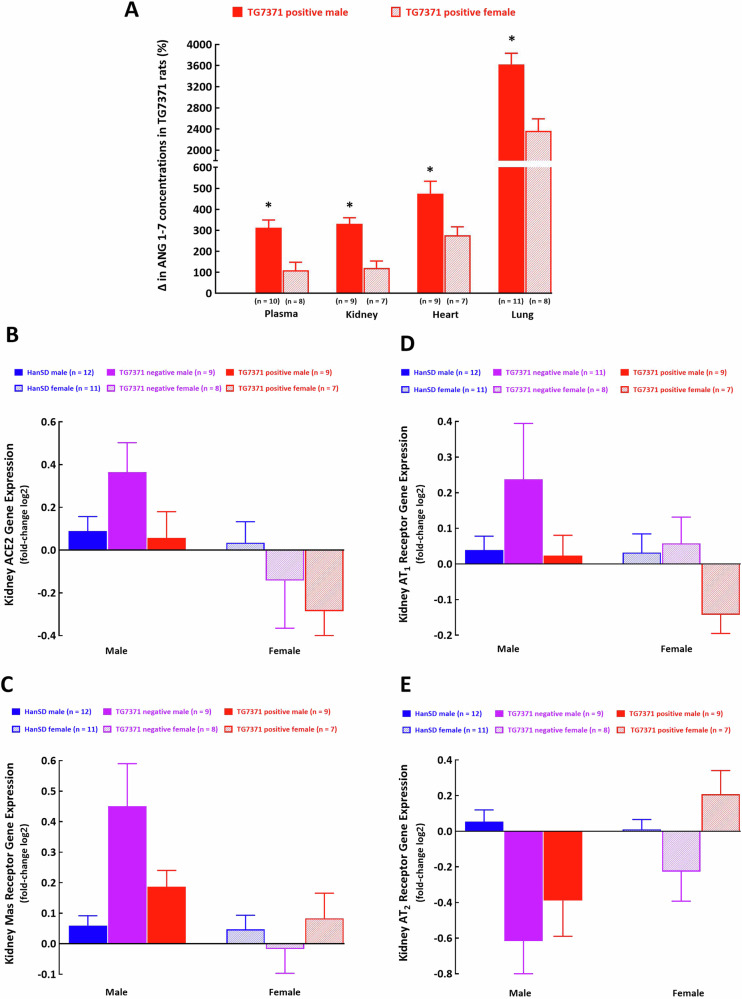
**A** Overview of percentage changes in angiotensin-(1-7) (ANG 1-7) concentration in male and female transgene-positive rats for ANG 1-7-producing fusion protein (TG7371 positive) versus Hannover Sprague-Dawley (HanSD) rats that served as 100% basal controls. Kidney mRNA gene expression of angiotensin-converting enzyme type 2 (ACE2) (**B**), Mas receptor (**C**), angiotensin II type 1 (AT_1_) receptor (**D**), an angiotensin II type 2 (AT_2_) receptor (**E**) in control HanSD rats (shown in blue), in transgene-negative rats for ANG 1-7-producing fusion protein (TG7371 negative) (violet) and in TG7371 positive (red). Data for males are shown as intense-colored bars and for females as light-colored bars

Below we present an overview of percentage changes in ANG 1-7 concentrations in male and female transgene-positive TG7371 versus male and female HanSD rats. The increases in ANG 1-7 concentrations in male TG7371 transgene-positive rats were more pronounced than in female TG7371 transgene-positive rats: for plasma the change was from +312 ± 37% to +109 ± 39%; in the kidney from +331 ± 29% to +121 ± 33%, in the heart LV from +475 ± 59% to +276 ± 41% and in the lung from +3624 ± 209% to +2364 ± 229% (Fig. 4A).

### Effects of expression of an ANG 1-7-producing fusion protein on kidney mRNA expression of ACE2, AT_1_, AT_2_ and Mas receptors

There were no significant differences between male and female TG7371 transgene-positive, TG7371 transgene-negative and HanSD rats in kidney mRNA expression of ACE2, AT_1_, AT_2_ and Mas receptors (Fig. 4B–E).

### Effects of expression of an ANG 1-7-producing fusion protein on SBP

**Fig. 5:**
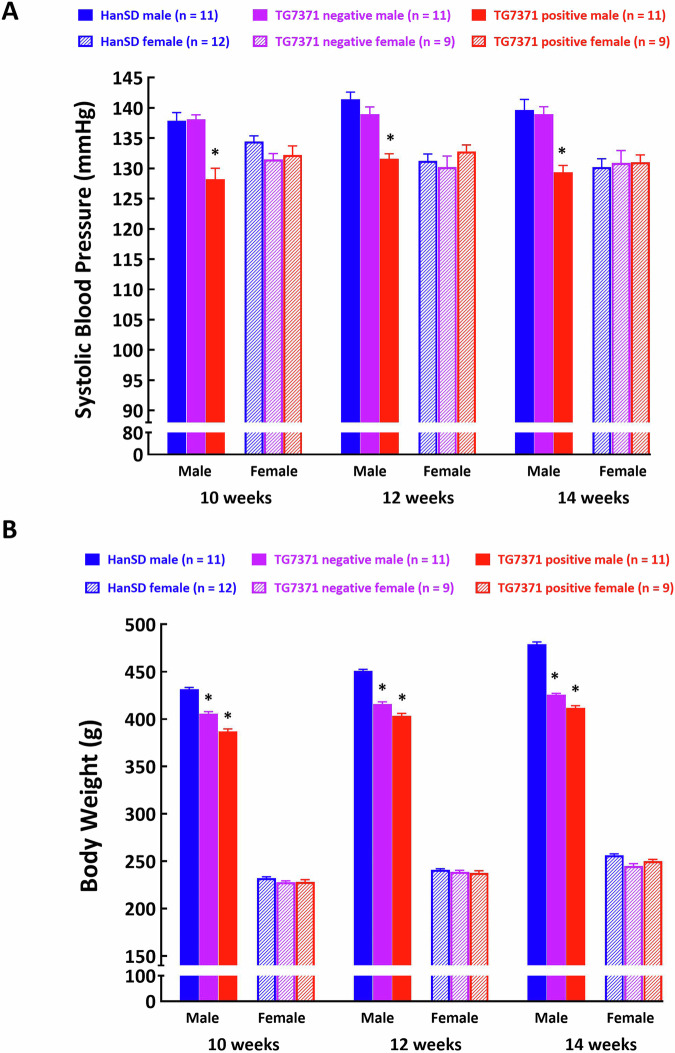
**A** Systolic blood pressure and **B** body weight in control Hannover Sprague-Dawley (HanSD) rats (shown in blue), in transgene-negative rats for ANG 1-7-producing fusion protein (TG7371 negative) (violet) and in transgene-positive rats for ANG 1-7-producing fusion protein (TG7371 positive) (red). Data for males are shown as intense-colored bars and for females as light-colored bars. ^*^P < 0.05 compared with HanSD rats, always at the same age

SBP in male TG7371 transgene-positive rats was significantly lower than in male TG7371 transgene-negative and male HanSD rats at the age of 10, 12 and 14 weeks (Fig. 5A). In contrast, there were no significant differences in SBP in female TG7371 transgene-positive, TG7371 transgene-negative and HanSD rats at any age (Fig. 5A). SBP did not significantly change with the age increasing from 10 to 14 weeks, and this was so in either strain (Fig. 5A).

Male TG7371 transgene-negative and TG7371 transgene-positive rats showed lower body weight as compared with male HanSD rats at the age of 10, 12 and 14 weeks (Fig. 5B). In contrast, there were no significant differences in body weight in female TG7371 transgene-positive, TG7371 transgene-negative and HanSD rats at any age (Fig. 5B).

## Discussion

The main objective of the present study was to characterize the systemic and organ activity of the classical as well as of the alternative arm of the RAS in a new transgenic rat strain, TG7371 transgene-positive rats, the strain with ubiquitous expression of an ANG 1-7-producing fusion protein. For this purpose, plasma and tissue concentrations of ANG II, the biologically most important peptide of the classical axis of the RAS, and ANG 1-7, the biologically most important peptide of the alternative axis of the RAS, were assessed in TG7371 transgene-positive rats, TG7371 transgene-negative rats and control HanSD rats of either sex. We found that male TG7371 transgene-positive rats exhibited significantly elevated plasma, kidney, heart LV and lung ANG 1-7 concentrations as compared with either TG7371 transgene-negative rats or with control HanSD rats. There were no significant differences in circulating and organ ANG II levels in male TG7371 transgene-positive rats, TG7371 transgene-negative rats and control HanSD rats. Therefore, the balance between the classical arm and the alternative arm of the RAS (expressed as the ANG 1-7 to ANG II ratio), both in the circulation and in all analyzed tissues, was in male TG7371 transgene-positive rats markedly shifted in favor of the alternative RAS arm. Activation of the alternative arm of the RAS in male TG7371 transgene-positive rats was associated with lower SBP, both compared with male TG7371 transgene-negative rats and with control male HanSD rats. In contrast, female TG7371 transgene-positive rats did not show activation of the alternative arm of the RAS (again assessed by the ANG 1-7 to ANG II ratio) in the circulation and in the kidney, and did not show significantly lower SBP as compared with both female TG7371 transgene-negative rats and with control female HanSD controls. These findings support the recently emerging notion, expressed by Alves et al. [21] and based on their comprehensive study in males, that this transgenic rat model might provide a new tool to investigate the long-term impact of increased circulatory and, in particular, tissue ANG 1-7 concentrations on the pathophysiology of cardio-renal and other diseases. However, even though the recent [21] and our present pertinent work support this concept, some of our findings expand our knowledge about this new model and deserve special consideration.

First, we showed here that the body weight of male TG7371 transgene-positive rats was significantly lower than that of male HanSD controls, despite the same age in both groups. Therefore, it is important to normalize the organ weights (particularly whole heart weight and left ventricle weight) to body weight in order to dismiss misleading information about organ weight changes which follow experimental manipulations. We cannot currently provide a reasonable explanation why male TG7371 transgene-positive rats as well as TG7371 transgene-negative rats showed consistently lower body weight as compared with male HanSD, even though both have the same genetic background. Evidently, future studies beyond our present scope are needed to address this issue.

Second, we found that female TG7371 transgene-positive rats showed less augmentation of the alternative axis of the RAS as compared with their male counterparts. Despite the apparent increasing tendency, plasma ANG 1-7 levels and the ANG 1-7/ANG II ratio were not significantly higher than the values observed in female control HanSD as well as in female TG7371 transgene-negative rats. Likewise, female TG7371 transgene-positive rats did not show significant elevation in the kidney ANG 1-7/ANG II ratio as compared with female control HanSD rats; this suggested that the state of intrarenal balance between the classical and the alternative axis of the RAS has not been shifted in favor of the latter. However, it is important to recognize that female TG7371 transgenic-positive rats showed markedly increased ANG 1-7 levels as well as the ANG 1-7/ANG II ratios in the lung and heart LV tissues, even though the increases were smaller than in their male counterparts. Taken together, our findings suggest that also female TG7371 transgenic-positive rats exhibit increased activation of the alternative axis of the RAS, but it is less pronounced than in male TG7371. In particular, the activity of this alternative axis of the RAS is not increased in the circulation and kidney of the female TG7371 transgenic-positive rats despite marked overexpression of ANG 1-7-producing fusion protein in the kidney. These interesting findings are in contradiction with the common view that all components of the alternative axis of the RAS exhibit greater expression and activation in females [15, 27].

We cannot currently provide a satisfactory explanation for the contrast between male and female TG7371 transgenic-positive rats regarding systemic and intrarenal activation of the alternative axis of the RAS; future studies are needed to address this issue. Nevertheless, our present findings showing that male TG7371 transgene-positive rats in contrast to female TG7371 transgene-positive rats showed marked systemic and intrarenal activation of the alternative axis of the RAS (accompanied by SBP lowering) without altering the classical arm of the RAS are in agreement with the concept about the critical role of systemic and particularly intrarenal RAS in the long-term BP control [1, 2, 4, 8, 45]. The novelty of our findings consists in demonstrating that the selective augmentation of the alternative (protective) arm of the RAS can itself lower BP. Since this has been seen in animals with normotensive background phenotype, it is of special interest as it implies that under some pathophysiological conditions, e.g. CKD or HF, augmentation of the activity of the alternative arm of the RAS could induce more pronounced protection; future studies are needed to evaluate this intriguing notion. Moreover, our results clearly show that the increase in plasma ANG 1-7 concentrations in male and female TG7371 transgenic-positive rats (as compared with control male and female HanSD rats) strictly correlates with increases in kidney ANG 1-7 concentrations (Fig. 4A). This implies that elevated plasma ANG 1-7 concentrations result from its spillover from the intrarenal compartment to the circulation, however, this idea again needs confirmation. Nevertheless, this still would not explain the discrepancy between our present findings showing markedly elevated plasma ANG 1-7 levels in male TG7371 transgenic-positive rats and the recent findings by Alves et al. [21]. Similarly, as in our study they reported normal plasma ANG II levels but also normal plasma ANG 1-7 levels (compared with HanSD rats). We do not have any satisfactory explanation for this discrepancy but it further underscores the need for more thorough phenotyping, including evaluation of basic renal function and cardiac function in this new very promising model. Only when this is accomplished, studies evaluating the role of the alternative arm of the RAS in the pathophysiology of cardio-renal diseases should follow and the studies of the therapeutic value of the protective arm of the RAS in cardiovascular diseases should start employing TG7371 transgenic-positive rats.

On the whole, our present findings also support the notion that preclinical research should be performed in both sexes [22, 26]. This would be critically important when employing the model with augmentation of the alternative axis of the RAS with an aim to study the pathophysiology of cardio-renal syndrome and chronic kidney diseases. In such conditions the state of the intrarenal balance between the two arms of the RAS might be decisively important [4, 5, 15, 16], and sex-linked differences are also well-known [10, 46–48].

Third, of particular interest are our findings regarding kidney mRNA expression of ACE2 and angiotensin receptors (Mas, AT_1_ and AT_2_ receptors): it was crucial to find out if chronic expression changes of ANG 1-7-producing fusion protein and persistently elevated ANG 1-7 levels do not alter this critically important enzyme and receptors of the RAS. Previous studies have demonstrated that ACE2 is dominantly responsible for the formation of ANG 1-7 [15, 49] and the focal overexpression of ACE2 in the rostral ventrolateral medulla or in the vascular smooth muscle lowered BP in spontaneously hypertensive rats (SHR) [50, 51]. Moreover, vascular overexpression of ACE2 was reported to protect the kidney against ageing-induced injury [52]. Since it is known that ACE2 produces ANG 1-7 from ANG II, many beneficial outcomes observed with overexpression of ACE2 can also be ascribed to a decrease in ANG II concentrations and not only to an increase in ANG 1-7 levels [15, 53]. However, our present findings show that ACE2 expression was not increased and ANG II concentrations were also not changed in TG7371 transgenic-positive rats. Therefore, all the effects observed should be ascribed to overexpression of an ANG 1-7-fusion protein and the resultant elevation of ANG 1-7 concentration. It is known that the majority of classical actions of ANG II, such as vasoconstriction, sodium retention, BP increase and stimulation of aldosterone release are mediated by AT_1_ receptor [1, 2, 8]. Since AT_1_ receptors in the kidney play a critical role in promoting hypertension and ANG II-dependent cardiac hypertrophy [45, 54], chronically elevated ANG 1-7 concentrations would alter kidney AT_1_ receptors expression. Notably, some actions of ANG 1-7 in TG7371 transgenic-positive rats could be also mediated by alterations in AT_1_ receptor activity. However, we showed here that the kidney AT_1_ receptor expression was not changed; it is therefore unlikely that any substantial influence of elevated ANG 1-7 concentration was mediated by modifications of AT_1_ receptor function in the kidney.

Historically and according to current textbook knowledge chronically elevated concentration of extracellular signaling molecules, such as norepinephrine and ANG II, by negative feedback causes down-regulation an appropriate receptors [17, 55, 56]. However, this view is not fully valid, at least not with regard to the RAS. It has been documented that in various forms of ANG II-dependent hypertension the increased ANG II concentration does not downregulate the renal AT_1_ receptors [57–60] and may even upregulate renal AT_1_ [61]. Similarly, it has been shown that pharmacologically induced elevation of ANG 1-7 levels does not decrease the expression of Mas receptors in SHR [62, 63]. It has even been found that vascular overexpression of ACE2 resulted in increased expression of Mas receptors in the renal vasculature [52]; this suggested that the ligand of Mas receptors, ANG 1-7, exerts a positive feedback action. Our present findings show that the expression of Mas receptors in the kidney did not differ between experimental groups and was not dependent on the sex. However, maintained kidney Mas receptor expression combined with exceptionally high intrarenal ANG 1-7 levels in male TG7371 transgenic-positive rats likely provides the basis for the sustained influence of ANG 1-7 on renal tubular reabsorption and consequently on BP. Admittedly, this notion requires further studies to confirm the potential physiological consequences of combined increased intrarenal ANG 1-7 levels and maintained Mas receptors expression in this new promising model.

Angiotensin AT_2_ receptors were discovered 35 years ago but their role was initially unclear. It is now recognized that together with ACE2/ANG 1-7/Mas receptor pathway, the activation of AT_2_ receptors contributes to the protective action of the RAS [64]. Besides ANG II, AT_2_ receptors are also activated by ANG 1-7 [64]. It was reported that long-term stimulation of ACE2/ANG 1-7/Mas axis resulted in downregulation of AT_2_ receptors [61]. Therefore, we also examined if the chronically elevated ANG 1-7 concentrations would alter kidney AT_2_ receptor expression: if so, it could diminish protective actions of ANG 1-7 in the TG7371 transgenic-positive rats. However, our present findings show that the kidney AT_2_ receptor expression was not changed so, it seems that in TG7371 transgenic-positive rats this part of the protective arm of the RAS is not altered.

Our fourth interesting finding is that ANG 1-7 concentration and ANG 1-7/ANG II ratio in TG7371 transgenic-positive rats is far more increased in the lung tissue than in the circulation, kidney or heart LV tissues. There is growing awareness that the alternative axis of the RAS is not only present in the former organs, but it might be important for regulation of their physiological functions and might influence the local pathophysiological processes [15, 53, 65]. Thus, the model with so greatly increased tissue activity of the alternative arm of the RAS in the lung might be extremely valuable for studies of its role in the pathophysiological processes in this organ, which is still a largely unexplored area.

### Limitations of the Study

Apart from the above-discussed reservations, two additional limitations of the present study should be considered.

The first is the discrepancy of opinions regarding the reliability of BP measurement. On one side, the editors of *Hypertension* assert that noninvasive tail-cuff measurements of BP is a fully acceptable method [37] and the invasive radiotelemetry method is not obligatory. On the other side, there is also a consensus that the noninvasive tail-cuff approach is fully reliable only if the difference in BP between groups or responses to an intervention exceed 10 mmHg or even 15 mmHg [37]. Since TG7371 transgene-positive rats were generated from animals with normotensive background phenotype, it is apparent that long-term studies employing radiotelemetry method will be required to unquestionably assess the effects of long-term activation of the alternative arm of the RAS on BP phenotype, and this notion is particularly valid in female TG7371 transgene-positive rats.

Second, since it is intended that TG7371 transgenic-positive rats serve as a model to study the role of long-term activation of the alternative arm of the RAS in the pathophysiology of cardio-renal diseases, more comprehensive analysis of kidney function (e.g. information on albuminuria, clearance of endogenous creatinine) and analysis of cardiac structure and functions (echocardiography and pressure-volume analysis) should be performed under basal conditions (i.e. before induction a pathophysiological insult.

## Conclusion

Notwithstanding all the aforementioned limitations, our present results together with the recent first phenotyping study [21] clearly demonstrate that male TG7371 transgenic-positive rats represent a model with selectively increased activity of the alternative axis of the RAS in the circulation as well as in all the critically important organs. Therefore, this new transgenic rat model is a new powerful tool to study the role of increased ANG 1-7 levels in the pathophysiology and potentially also in the treatment of cardio-renal and possibly also other diseases.
